# Design and facile synthesis of defect-rich C-MoS_2_/rGO nanosheets for enhanced lithium–sulfur battery performance

**DOI:** 10.3762/bjnano.10.217

**Published:** 2019-11-14

**Authors:** Chengxiang Tian, Juwei Wu, Zheng Ma, Bo Li, Pengcheng Li, Xiaotao Zu, Xia Xiang

**Affiliations:** 1 School of Physics, University of Electronic Science and Technology of China, Chengdu 610054, China

**Keywords:** annealing, double modification, high-performance electrodes, lithium–sulfur battery, molybdenum disulfide (MoS_2_), reduced graphene oxide (rGO)

## Abstract

We report a simple one-step hydrothermal strategy for the fabrication of a C-MoS_2_/rGO composite with both large surface area and high porosity for the use as advanced electrode material in lithium–sulfur batteries. Double modified defect-rich MoS_2_ nanosheets are successfully prepared by introducing reduced graphene oxide (rGO) and amorphous carbon. The conductibility of the cathodes can be improved through the combination of amorphous carbon and rGO, which could also limit the dissolution of polysulfides. After annealing at different temperatures, it is found that the C-MoS_2_/rGO-6-S composite annealed at 600 °C yields a noticeably enhanced performance of lithium–sulfur batteries, with a high specific capacity of 572 mAh·g^−1^ at 0.2C after 550 cycles, and 551 mAh·g^−1^ even at 2C, much better than that of MoS_2_-S nanosheets (249 mAh·g^−1^ and 149 mAh·g^−1^) and C-MoS_2_/rGO-S composites (334 mAh·g^−1^ and 382 mAh·g^−1^). Our intended electrode design protocol and annealing process may pave the way for the construction of other high-performance metal disulfide electrodes for electrochemical energy storage.

## Introduction

Lithium–sulfur (Li–S) batteries have attracted great attention because of the high energy density (2600 Wh kg^−1^) and specific capacity (1675 mAh·g^−1^), low cost, and abundant reserves of elemental sulfur [1–2]. Nevertheless, there are various technical challenges in the development of Li–S batteries. The intrinsic insulation properties of the discharge products (Li_2_S_2_ and Li_2_S) and sulfur result in a slow charge and discharge process and a low specific capacity [3]. Intermediate products of battery charge and discharge (Li_2_S*_n_*, where 3 ≤ *n* ≤ 8) are soluble in the electrolyte and can also migrate to the lithium metal anode and precipitate there [4–5]. The decay of the electrochemically active lithium polysulfides causes rapid capacity degradation during charge and discharge process.

In order to overcome the problems above, great efforts have been made to improve the performance of Li–S batteries, including combining conductive materials with sulfur [6–8], constructing Li_2_S*_n_* blocking interlayers [9–11], and applying functional separators [12–15]. Although there are many methods, the most common strategy is to combine sulfur with various carbon materials owing to their excellent conductivity and flexible nanostructures. However, the capacity of carbon and sulfur composite cathodes generally fades rapidly during long-term cycling, because the carbon materials can provide only inferior physical adsorption to the polar Li_2_S*_n_* [16]. Once Li_2_S*_n_* is solvated, it dissolves easily in the electrolyte from the electrode surface and subsequently disperses. Consequently, the reutilization of Li_2_S*_n_* will become very hard due to the repulsion between the nonpolar conductive surface and the polar reactants [17].

Two-dimensional layered transition metal dichalcogenides (TMDs), strong candidates in the search for energy storage and catalyst materials, can provide good performance at low cost [18–20]. In particular, MoS_2_ has attracted the most attention owing to the high electrochemical activity associated with the sulfur deficiencies [5]. It has been reported that MoS_2_ nanosheets show great performance in the hydrodesulphurization process catalyzing the formation of sulfur species [21–22], indicating a potential application for Li–S batteries. Previous simulation results show that the binding energy of the edge active sites of defect-rich MoS_2_ and Li_2_S is much greater than the binding energy of the base plane, which is very helpful for the adsorption of polysulfides [23–24]. However, the low conductivity of MoS_2_ often results in incomplete conversion of polysulfides. Thus, MoS_2_ is usually combined with carbon materials and annealing treatment is also considered [25–26]. Hence, double modification of defect-rich MoS_2_ nanosheets with amorphous carbon and rGO followed by thermal annealing should be a very hopeful strategy to increase the performance of sulfur cathodes.

In this work, we firstly present a double carbon network modification method for defect-rich MoS_2_ anodes by introducing amorphous carbon and rGO via a one-step hydrothermal method. We concentrate not only on the material design based on both structure and chemical composition but also on the development of MoS_2_ electrocatalysts. Firstly, the MoS_2_ nanosheets are interconnected with rGO and then well covered by the carbon layer. This means that overall connected conductive networks are formed by the combination of amorphous carbon layer and rGO. Secondly, the existence of a great number of defects in the ultrathin MoS_2_ nanosheets leads to partial breaking of the catalytically inert basal planes, yielding additional active edge sites. Thirdly, annealing of the C-MoS_2_/rGO composites at different temperatures has also been investigated. The annealing can improve crystallinity and increase the binding energy, and also improve the stability while maintaining high specific capacity. The defect-rich C-MoS_2_/rGO prepared in this work can not only kinetically accelerate the sulfur redox reactions but also chemically adsorb polysulfides. In addition, rGO and carbon layer can also enhance the conductivity of C-MoS_2_/rGO. Therefore, the C-MoS_2_/rGO, as an efficient sulfur host, could exhibit excellent electrochemical performance.

## Experimental

### Preparation of defect-rich C-MoS_2_/rGO nanosheets

For the synthesis of the C-MoS_2_/rGO nanosheets, 1.23 g hexaammonium heptamolybdate tetrahydrate, 0.89 g of glucose, and 2.28 g thiourea were dissolved in 25 mL of deionized water to form solution A; 0.03 g of GO was dissolved into 10 mL of deionized water by ultrasonic to form solution B. Then solution A is mixed with solution B and stirred for 30 min. Subsequently, the mixture was transferred to a 50 mL Teflon-lined stainless steel autoclave and retained at 200 °C for 24 h. After cooling naturally, the product was washed with deionized water and absolute ethanol for several times and dried at 60 °C in vacuum. For comparison, the pristine MoS_2_ were synthesized using an identical process without GO or glucose. After that, the obtained composites were annealed at 400, 600 and 800 °C for 6 h in 10% H_2_/Ar atmosphere to improve the crystallinity. After thermal annealing, the samples were labeled as C-MoS_2_/rGO-4, C-MoS_2_/rGO-6, and C-MoS_2_/rGO-8, respectively.

### Preparation of sulfur composites

MoS_2_-S, C-MoS_2_/rGO-S, C-MoS_2_/rGO-4-S, C-MoS_2_/rGO-6-S, C-MoS_2_/rGO-8-S composites were fabricated by melt diffusion. The mixture of sulfur and pristine MoS_2_, C-MoS_2_/rGO and annealed composites (C-MoS_2_/rGO-4, C-MoS_2_/rGO-6, C-MoS_2_/rGO-8) with a 3:1 mass ratio was calcined at 155 °C for 12 h in a sealed vessel.

### Materials characterization

The structure and morphology were investigated by field-emission scanning electron microscopy (FE-SEM, INSPECT F50) and transmission electron microscopy (TEM, ZEISS Libra 200). Powder X-ray diffraction (XRD) measurements were conducted to determine the phase of the as-synthesized composites with Cu Kα radiation operated at 40 kV and 30 mA. X-ray photoelectron spectroscopy (XPS) analysis was performed on a Kratos AXIS Ultra DLD instrument using monochromated Al Kα X-rays as the excitation source. Raman spectra were collected using a Witec alpha 300M+ instrument with an excitation laser wavelength of 488 nm. Nitrogen adsorption–desorption isotherm measurements were conducted at 77 K using a micromeritics system (JW-BK132F). The contents of amorphous carbon, rGO and sulfur in the samples were analyzed by thermogravimetric (TGA) on a Netzsch STA 449C analyzer in air for the amorphous carbon and rGO or in N_2_ atmosphere for the sulfur at a temperature ramp rate of 10 °C·min^−1^.

### Lithium polysulfide adsorption tests

The concentration of the fabricated Li_2_S_6_ solution was set to 3 mM by dissolving lithium sulfide (Li_2_S) and sulfur with a molar ratio of 1:5 in 1,3-dioxolane (DOL) and dimethoxymethane (DME) (1:1 by volume) and then stirring for overnight in a glovebox. After that, 5 mg of pristine MoS_2_, C-MoS_2_/rGO and annealed composites were added to the Li_2_S_6_ solution (3 mL) as the adsorbents. Ultraviolet–visible (UV–vis) absorption spectra of these diluted solutions were collected using a Shimadzu UV 2550 spectrophotometer.

### Cell assembly and electrochemical measurements

The working electrodes were prepared by casting a slurry of 80 wt % active materials (MoS_2_-S, C-MoS_2_/rGO-S, C-MoS_2_/rGO-4-S, C-MoS_2_/rGO-6-S, C-MoS_2_/rGO-8-S), 10 wt % acetylene black and 10 wt % polyvinylidene fiuoride (PVDF) in *N*-methyl-2-pyrrolidone (NMP) on an Al foil current collector. Then, the electrodes were dried in vacuum at 60 °C for 12 h.

The electrode was manufactured in a coin-type cell (CR 2032) in an argon-filled glove box (O_2_ < 0.1 ppm, H_2_O < 0.1 ppm). The electrolyte was 1 M bis(trifluoromethane) sulfonimide lithium salt (LiTFSI) dissolved in a mixed solution of dimethyl ether (DME) and 1,3-dioxolane (DOL) (1:1, v/v) with 2 wt % LiNO_3_. The recharge properties and cyclic voltammetry tests were carried out on a LAND battery cycler (CT2001A) in a voltage range of 1.7 to 2.9 V (vs Li/Li^+^). The specific capacity was calculated based on the mass of sulfur. Cyclic voltammetry (CV) tests were carried out between 1.6 and 2.9 V at a scan rate of 0.1 mV·s^−1^. The electrochemical impedance spectroscopy (EIS) measurements were achieved at the open-circuit potential between 0.01 Hz and 100 kHz. All the tests were carried out at room temperature.

## Results and Discussion

**Figure 1:**
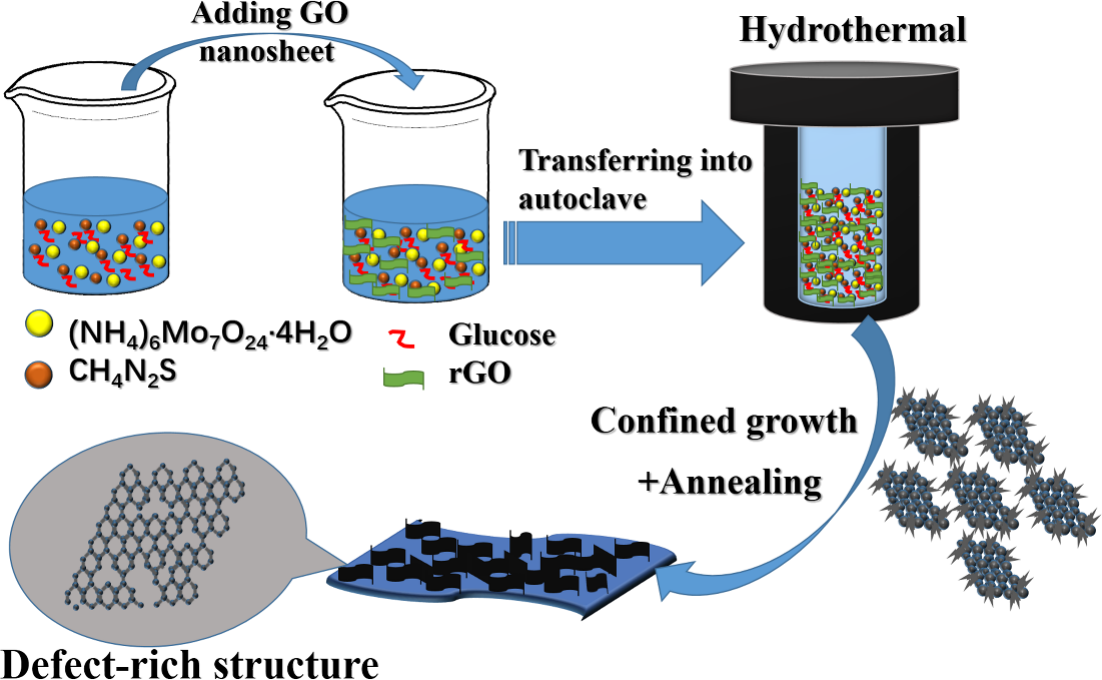
Schematic illustration of the synthesis of C-MoS_2_/rGO composite.

The preparation of the C-MoS_2_/rGO composite is shown in Figure 1. The C-MoS_2_/rGO composite is synthesized by the simple and efficient hydrothermal route followed by annealing.

**Figure 2:**
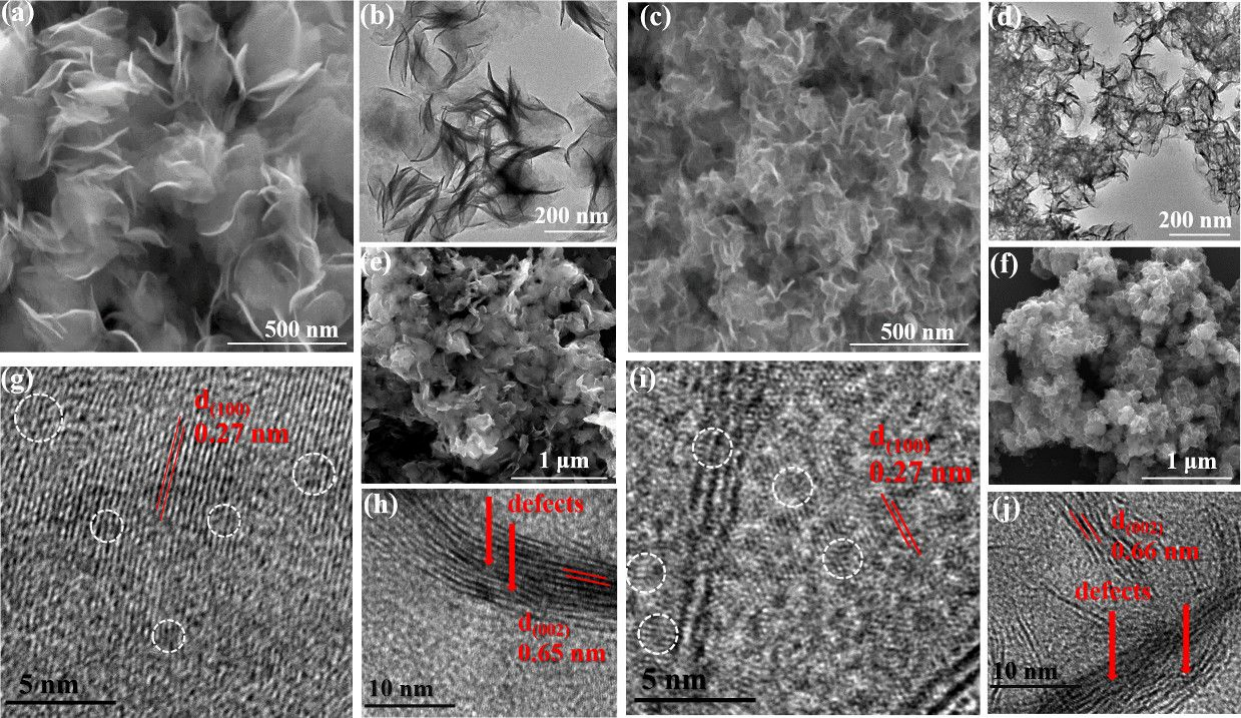
(a) SEM and (b) TEM images of pristine MoS_2_; (c) SEM and (d) TEM images of C-MoS_2_/rGO; (e) SEM image of MoS_2_-S and (f) SEM image of C-MoS_2_/rGO-S; (g, h) HRTEM images of pristine MoS_2_ and (i, j) HRTEM images of C-MoS_2_/rGO.

The SEM image in Figure 2a clearly reveals the morphology of the pristine ultrathin MoS_2_ nanosheets. The apparent corrugations and ripples can be shown and the lateral size of the nanosheets is 200–300 nm. A corresponding TEM image also verifies the ultrathin nanosheet morphology, as shown in Figure 2b. Due to the addition of amorphous carbon and rGO, the size of C-MoS_2_/rGO nanosheets obviously becomes smaller, as shown in Figure 2c,d. After melting with elemental sulfur, the MoS_2_ nanosheets keep the sheet-like morphology as shown in Figure 2e,f. The morphology of nanosheets does not change even after annealing at 800 °C, as shown in Figure S1a–d (Supporting Information File 1).

An interplanar spacing of 0.27 nm can be observed in Figure 2e and Figure 2g. Numerous distortions and dislocations can be observed, which indicates a defect-rich structure as shown in the white circles in Figure 2e and Figure 2g. A similar phenomenon is also observed for C-MoS_2_/rGO-4 and C-MoS_2_/rGO-8 as shown in Figure S1e and Figure S1g (Supporting Information File 1). The additional edges are generated by breaking of the basal planes due to disordered atomic arrangement, which is beneficial for catalyzing the formation of polysulfides. Furthermore, the typical lamellar structure with interlayer spacings of 0.65 nm and 0.66 nm can be observed in Figure 2f and Figure 2h; the layer-to-layer spacing is slightly larger than the spacing of 6.2 nm in bulk MoS_2_ [27–29]. The crystal nanosheets along the edge are irregular, which can also be ascribed to the successful introduction of rich defects, as indicated by the arrows in the images. Therefore, a better energy storage performance may be anticipated from the double modified defect-rich MoS_2_ nanosheets.

**Figure 3:**
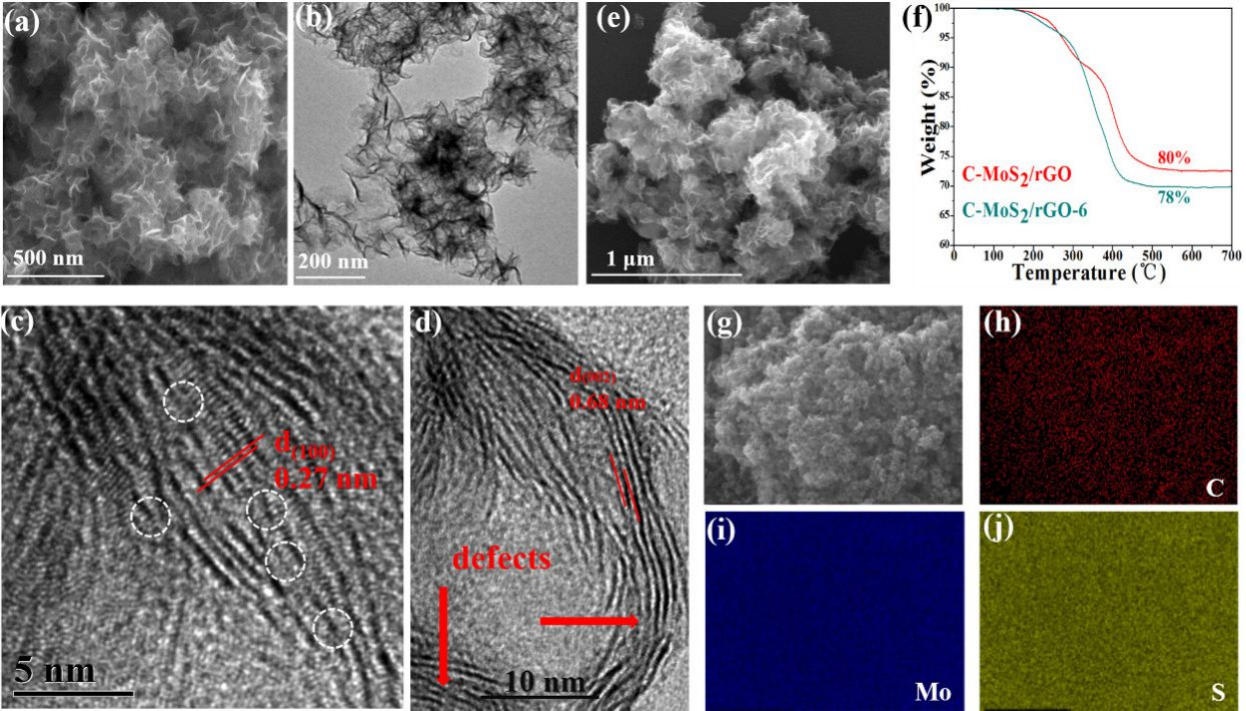
Morphological images of the annealed C-MoS_2_/rGO-6 composite: (a) SEM; (b) TEM; (c, d) HRTEM; (e) SEM image of C-MoS_2_/rGO-6-S; (f) TG analysis curve; (g–j) element mapping images of Mo, S, and C.

After annealing, the lattice fringes distance is further increased to about 0.68 nm, and the fringes of the edges are also apparently curled as shown in Figure 3a–d. The lattice fringes distance does not change even after annealing at 800 °C, as shown in Figure S1f and Figure S1h (Supporting Information File 1). The sheet-like morphology of the C-MoS_2_/rGO-6-S composite can still be observed as shown in Figure 3e. A typical TG analysis (Figure 3f) is performed in atmosphere from room temperature to 800 °C. MoS_2_ and carbon are oxidized to MoO_3_ and CO_2_ in this temperature range [3,30].

After calculation, the mass fractions of MoS_2_ in the C-MoS_2_/rGO and C-MoS_2_/rGO-6 composites are about 80 wt % and 78 wt %, respectively. The content decrease of C-MoS_2_/rGO-6 composite may be due to decomposition of MoS_2_ in 10% H_2_/Ar atmosphere during annealing when the temperature is higher than 600 °C [31–32]. The homogeneous diffusion of the elements is confirmed by the selected element mappings as shown in Figure 3g–j.

**Figure 4:**
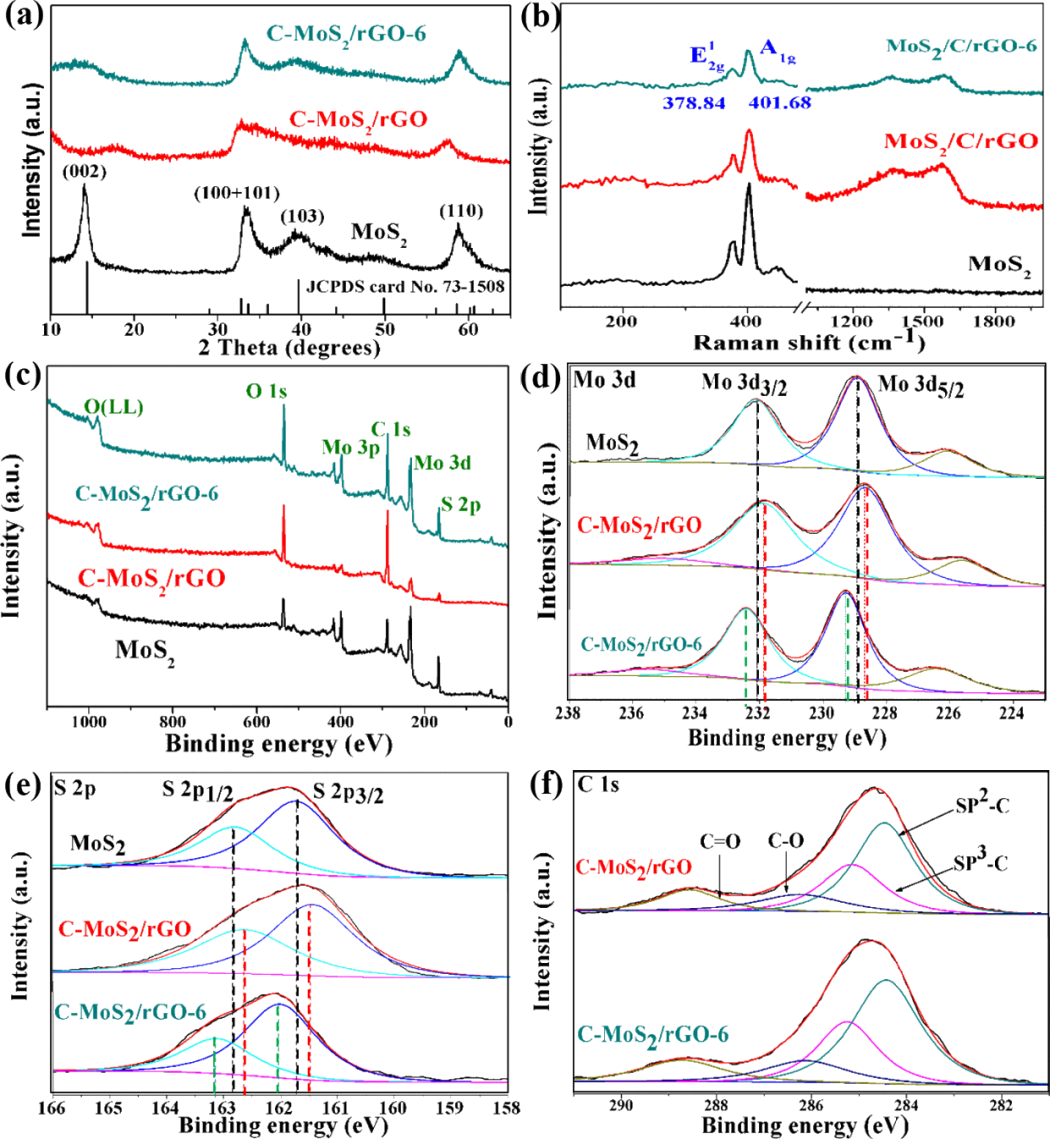
(a) XRD patterns, (b) Raman spectra, (c) full scan XPS spectra and high-resolution XPS spectra of (d) Mo 3d and (e) S 3d of pristine MoS_2_, C-MoS_2_/rGO and C-MoS_2_/rGO-6 composites, and (f) high-resolution XPS spectra of C 1s C-MoS_2_/rGO and C-MoS_2_/rGO-6 composites, respectively.

Figure 4a shows the XRD patterns of pristine MoS_2_, C-MoS_2_/rGO and annealed C-MoS_2_/rGO-6 composites. For pristine MoS_2_, the diffraction peaks can be well indexed to the 2H-MoS_2_ phase (JCPDS 73-1508). The defect-rich structure causes the formation of smaller nanosheets along the basal planes in Figure 2e, which is consistent with the remarkable broadening of the (110) and (100) diffraction peaks. The peak at about 14.39°, which corresponds to the (002) planes, is shown in the XRD pattern of pristine MoS_2_ but not in that of the C-MoS_2_/rGO and C-MoS_2_/rGO-6 composite. This is ascribed to carbon layers and rGO nanosheets destroying the stacking of MoS_2_ layers, leading to the vanishing of these characteristic peaks. An analogous phenomenon has been described in other works [33–34]. The broad and weak peak at 17.65° for the C-MoS_2_/rGO composite might be attributed to the spacing between the MoS_2_ nanosheets and carbon layers [35–36], and it disappears after annealing. Compared with C-MoS_2_/rGO, it is noticeable that the diffraction peaks of the C-MoS_2_/rGO-6 become sharper, which also indicates that the annealing treatment improves the crystallinity of the composites.

Figure 4b and Figure S2 (Supporting Information File 1) show the Raman spectra of pristine MoS_2_, and C-MoS_2_/rGO, C-MoS_2_/rGO-6, C-MoS_2_/rGO-4 and C-MoS_2_/rGO-8 composites. Two clear peaks at about 378 cm^−1^ and 404 cm^−1^ show in all curves of MoS_2_, consistent with the in-plane 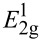 and out-of-plane *A*_1g_ vibrations of pristine MoS_2_, respectively [37]. In addition to these peaks from pristine MoS_2_, the Raman spectra of C-MoS_2_/rGO, C-MoS_2_/rGO-6, C-MoS_2_/rGO-4 and C-MoS_2_/rGO-8 exhibit two broad bands at 1363 cm^−1^ (D-band) and 1587 cm^−1^ (G-band) resulting from in-plane vibrations and defect-induced vibrations of sp^2^-hybridized carbon atoms in amorphous carbon and rGO [30].

The surface elements of the composites are analyzed by XPS. The survey spectrum confirms that the expected elements in the composites are C, Mo, O, and S (Figure 4c). The two peaks of pristine MoS_2_ at 228.9 and 232.1 eV in Figure 4d are assigned to binding energies of Mo 3d_5/2_ and 3d_3/2_ for Mo^4+^, respectively, corresponding to the 2H phase of MoS_2_[20]. The two peaks at 162.78 and 161.7 eV are attributed to the S 2p_1/2_ and S 2p_3/2_ orbital of divalent sulfide ions (S^2−^) corresponding to the 2H phase of MoS_2_, as shown in Figure 4e [38]. In general, MoS_2_ nanosheets with both 1T and 2H phases can be used as energy storage materials and these two phases often co-exist in the MoS_2_ samples. Usually, the metallic 1T phase of MoS_2_ contributes to enhanced conductivity and catalytic activity [13,25] while the defect-rich 2H phase of MoS_2_ has catalytic effects on polysulfides [3]. In this work, we focus on the defect-rich phase MoS_2_. Therefore, the 2H phase of MoS_2_ was prepared by designing a reaction with a high concentration of precursors and excess thiourea in order to utilize its defect sites to catalyze the conversion of polysulfides and to improve the energy storage performance. The binding energies of Mo 3d_5/2_ and Mo 3d_3/2_ in C-MoS_2_/rGO composite shift to 228.6 and 231.8 eV, respectively (Figure 4d). Similarly, the binding energies of S 2p_3/2_ and S 2p_1/2_ in the C-MoS_2_/rGO composite shift to lower energies by about 0.2 eV relative to pristine MoS_2_. This can be attributed to the interactions of MoS_2_, amorphous carbon and rGO, which weakens the binding energy of Mo and S [39]. However, after annealing, the binding energies of Mo 3d_5/2,_ Mo 3d_3/2,_ S 2p_3/2_ and S 2p_1/2_ in the C-MoS_2_/rGO-6 composite increase by about 0.4 and 0.3 eV, respectively, relative to those of pristine MoS_2_. The increased binding energy of C-MoS_2_/rGO-6 may due to the improved crystallinity after annealing, which is consistent with the results of XRD. These results strongly suggest the existence of electronic interactions between amorphous carbon and MoS_2_, which implies the establishment of coupling interfaces [40]. This interaction not only improves the rate of catalytic polysulfide conversion but also effectively utilizes the conductivity of carbon, which is the goal of designing materials that overcome the inherent defects of lithium–sulfur batteries.

The high-resolution C 1s spectrum can be deconvoluted into four peaks, as shown in Figure 4f. The peak at 284.5 eV with high intensity indicates that most C atoms in the composites can be assigned to sp^2^-hybridized carbon [33]. The peaks located at 285.1 and 286.2 eV correspond to the binding energy of carbon in C–C bonds and C–O bonds, respectively, owing to an amorphous carbon layer originated from glucose. The two weak peaks originating from C–O and C=O bonds illustrate that the oxygen-containing functional groups are almost completely removed during the hydrothermal synthesis and annealing process [41].

**Figure 5:**
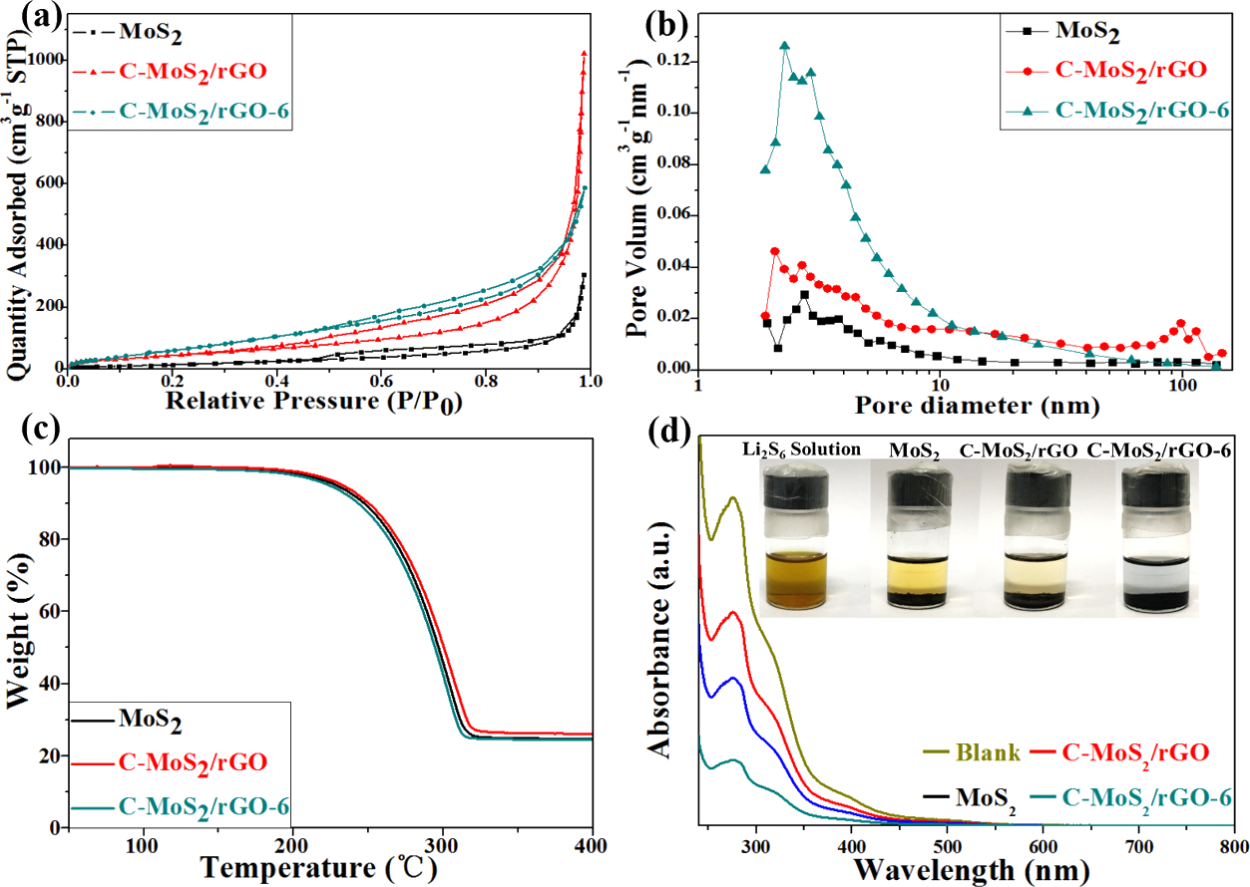
(a) N_2_ adsorption–desorption isotherms and (b) corresponding pore size distributions of pristine MoS_2_, and C-MoS_2_/rGO and C-MoS_2_/rGO-6 composites. (c) TG curves of MoS_2_-S, C-MoS_2_/rGO-S and C-MoS_2_/rGO-6-S composites; (d) UV–vis absorption spectra of the pure Li_2_S_6_ solution and Li_2_S_6_ solutions with pristine MoS_2_, and C-MoS_2_/rGO and C-MoS_2_/rGO-6 composites (inset: photos of the Li_2_S_6_ solution after 12 h of adsorption experiments with the same amount of pristine MoS_2_, or C-MoS_2_/rGO and C-MoS_2_/rGO-6 composites).

Full nitrogen sorption isotherms of the composites were measured to obtain the specific surface area and the pore size distribution. A type-IV isotherm with a type-H3 hysteresis loop in the relative pressure range of 0.45–1.0 *P*/*P*_0_ suggests the presence of a mesoporous structure, as displayed in Figure 5a.

The specific surface area was calculated to be 131.72, 300.64, 539.16 m^2^·g^−1^ by using the Brunauer–Emmett–Teller (BET) method. The pore size distribution obtained from the Barrett–Joyner–Halenda (BJH) method is given in Figure 5b. This difference can be ascribed to the nanostructure of smaller MoS_2_ nanosheets and the influence of amorphous carbon and rGO. The obtained high specific surface area of the C-MoS_2_/rGO-6 composite is mainly attributed to the following two aspects: a large amount of amorphous carbon is reduced by hydrogen to cause further increase of carbon defects; and the crystallinity of the MoS_2_ nanosheets is further improved. This is consistent with the results of XRD and Raman. The pore size distribution of the composites exhibits a sharp peak at 3 nm and another broad peak at 40 nm, demonstrating the mesoporous structure C-MoS_2_/rGO-6. The specific surface area (130.57 m^2^·g^−1^) of C-MoS_2_/rGO-6-S composite is significantly smaller than that of C-MoS_2_/rGO-6 as shown in Figure S3a (Supporting Information File 1). Additionally, the broad pore size distribution, ranging from 2 to 10 nm, diminished obviously in the C-MoS_2_/rGO-6-S composite (Figure S3b, Supporting Information File 1) compared to the C-MoS_2_/rGO-6 composite, which indicates that sulfur can be successfully dispersed into the mesoporous of the C-MoS_2_/rGO-6 composite. Among all samples, the C-MoS_2_/rGO-6 composite has the largest specific surface area.

The sulfur contents in the different composites as tested by TGA were about 75 wt % as shown in Figure 5c. The capability to adsorption polysulfides of pristine MoS_2_, C-MoS_2_/rGO and C-MoS_2_/rGO-6 was also measured as shown in Figure 5d. From the inset photograph, C-MoS_2_/rGO-6 composite renders the Li_2_S_6_ solution almost completely colorless while the solution of pristine MoS_2_ exhibits a stronger coloration than all solutions of C-MoS_2_/rGO composites. This could be attributed to the porous structure and high specific surface area of the C-MoS_2_/rGO-6 composite. The UV–vis absorption spectra of the four solutions were also investigated to better understand the adsorption capacity. Li_2_S_6_ in solution is still not completely absorbed by the C-MoS_2_/rGO-4 and C-MoS_2_/rGO-8 composites as shown in Figure S4a (Supporting Information File 1). Consequently, the colorless solution of C-MoS_2_/rGO-6 suggests that C-MoS_2_/rGO-6 exhibits the best polysulfide adsorption capability.

**Figure 6:**
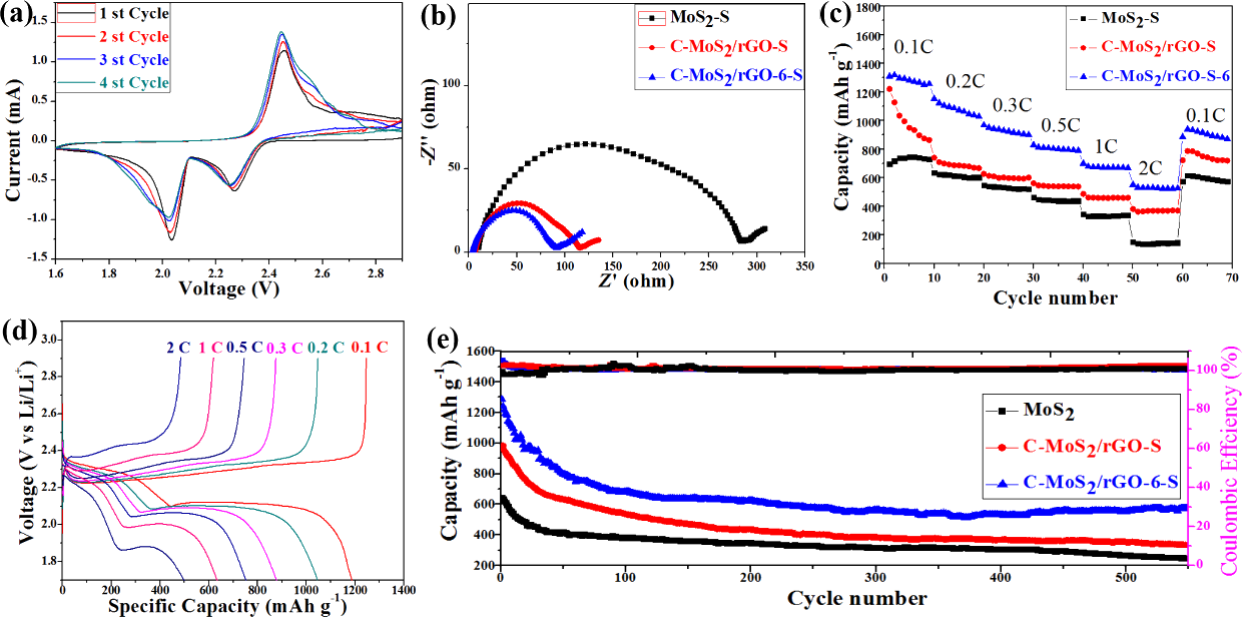
(a) Typical CV curves of the C-MoS_2_/rGO-6-S (b) Nyquist plots of MoS_2_-S, C-MoS_2_/rGO-S and C-MoS_2_/rGO-6-S composites over a frequency range 100 kHz to 0.01 Hz. (c) Comparison of rate capabilities at different C-rates for MoS_2_-S, C-MoS_2_/rGO-S and C-MoS_2_/rGO-6-S composites (d) Charge/discharge curves of C-MoS_2_/rGO-6-S at varied C rates (e) Discharge capacities and columbic efficiency for MoS_2_-S, C-MoS_2_/rGO-S and C-MoS_2_/rGO-6-S composites at a rate of 0.2C.

In order to illustrate the better electrochemical performance of the composites, three groups of samples, MoS_2_-S, C-MoS_2_/rGO-S, C-MoS_2_/rGO-6-S, were used as cathodes and evaluated using CR2032 coin cells. The obvious redox peaks show the multiple reaction mechanisms of sulfur cathodes in Li–S batteries (Figure 6a). Two explicit reduction peaks located at 2.25 V and 2.02 V correspond to the cathodic scanning. The peak at 2.25 V is related to the reduction of cyclic S_8_ to Li_2_S*_n_* and the peak at 2.02 V corresponds to the reduction of Li_2_S*_n_* to Li_2_S and Li_2_S_2_ [25,42]. Simultaneously, there is only one clear oxidation peak at 2.45 V corresponding to the anodic scanning, which is related to oxidation process of Li_2_S/Li_2_S_2_ to Li_2_S*_n_* [43]. After two cycles, the CV peak positions of the third and fourth cycles nearly overlap, indicating the superior electrochemical reversibility of the C-MoS_2_/rGO-6-S cathode.

The electrochemical impedance spectroscopy results of MoS_2_-S, C-MoS_2_/rGO-S, C-MoS_2_/rGO-6-S electrodes are shown in Figure 6b. Obviously, the C-MoS_2_/rGO-S and C-MoS_2_/rGO-6-S electrodes have the minor semicircles in the high-frequency range, suggesting a smaller charge transfer resistance than MoS_2_-S. Since every cathode holds the same amount of sulfur, the distinct charge transfer resistance could be ascribed to the conductivity of the different host materials. Benefiting from the synergistic encapsulation of the polar MoS_2_ nanosheets and conductive amorphous carbon and rGO, C-MoS_2_/rGO-6-S shows stronger chemical interaction with Li_2_S*_n_* and a higher ability to assist the charge transfer than the MoS_2_-S and C-MoS_2_/rGO-S electrodes.

Furthermore, the rate capabilities of the MoS_2_-S, C-MoS_2_/rGO-S, C-MoS_2_/rGO-6-S electrodes were measured in the galvanostatic mode at several charge–discharge rates, and the corresponding calculated capacities are shown in Figure 6c and Figure S4b (Supporting Information File 1). The C-MoS_2_/rGO-6-S electrode exhibits a higher capacity than the MoS_2_-S, C-MoS_2_/rGO-S, C-MoS_2_/rGO-4-S and C-MoS_2_/rGO-8-S electrodes in the total charge–discharge process. A discharge capacity of 694, 1221, 1309, 1142 and 734 mAh·g^−1^ is, respectively, achieved for the MoS_2_-S, C-MoS_2_/rGO-S, C-MoS_2_/rGO-6-S, C-MoS_2_/rGO-4-S, and C-MoS_2_/rGO-8-S electrodes after the first cycle at 0.1C. The C-MoS_2_/rGO-6-S electrode still shows a reversible capacity of 551 mAh·g^−1^ even at the elevated discharge and charge rate of 2C, indicating a better rate capability. A capacity of 939 mAh·g^−1^ is retained for the C-MoS_2_/rGO-6-S electrode, i.e., approximately 72% capacity retention of the initial capacity after the rate was adjusted back to 0.1C. In contrast, the MoS_2_-S and C-MoS_2_/rGO-S electrodes indicate noticeably lower discharge capacities in the range from 0.1 to 2C. For the MoS_2_-S, this is ascribed to its lower electronic conductivity. Compared with C-MoS_2_/rGO-S, the significant improvement of the capacity of the C-MoS_2_/rGO-6-S electrode can be attributed to the annealing, which does not only reduce amorphous carbon and GO but also enhances synergistic effects and results in additional powerful polar–polar chemical interactions rather than only weak physical adsorption of the polysulfides [8]. The galvanostatic discharge–charge curves of C-MoS_2_/rGO-6-S at several current densities are shown in Figure 4d. The two standard discharge plateaus can still be identified even at rate of 2C, indicating the effortless kinetics of the sulfur redox reaction.

Figure 6e and Figure S4c (Supporting Information File 1) compare the cyclic performances of MoS_2_-S, C-MoS_2_/rGO-S, C-MoS_2_/rGO-6-S, C-MoS_2_/rGO-4-S, and C-MoS_2_/rGO-8-S at 0.2 C. All samples show a rapid decay in the initial stage. The C-MoS_2_/rGO-6-S composite exhibits the largest initial specific capacity due to the large number of mesopores. The mesopores can hold more electrolyte to yield a good coverage of sulfur on the surface. Thus, more sulfur can react in the charge and discharge process during the initial several cycles. However, since the charge and discharge reaction causes a part of the polysulfide ions to dissolve and continuously enter the electrolyte, the sulfur is also continuously depleted. Consequently, a rapid decay of the specific capacity occurs when the composite is loaded with less sulfur. After several cycles of charge and discharge, the dissolved polysulfide ions in the electrolyte will reach a dynamic equilibrium. The dissolution of polysulfide ions into the electrolyte will be gradually restrained during the subsequent charge and discharge reaction. Therefore, the reduction rate of the specific capacity is lowered.

According to the literature [25–26,44], the combination of MoS_2_ and carbon materials can significantly improve energy storage performance. In this work, the as-synthesized C-MoS_2_/rGO-S electrodes did not show a clear improvement compared with pristine MoS_2_. This may be attributed to the higher concentration of the precursor during the synthesis process, insufficient carbonation of glucose and relatively poor crystallinity. However, the annealed C-MoS_2_/rGO-S samples significantly improve the performance. It is worth noting that the initial discharge specific capacities of MoS_2_, C-MoS_2_/rGO, and C-MoS_2_/rGO-6 composites are 643, 975, and 1285 mAh·g^−1^, respectively, and the specific capacities after 550 cycles are 249, 334, and 572 mAh·g^−1^, respectively. The C-MoS_2_/rGO-6 composite exhibits the best performance, which is consistent with the results of specific surface area testing and adsorption experiments. This also shows that the combination of MoS_2_, amorphous carbon and rGO not only has an ultra-high specific surface area but also enhances the conductivity. At the same time, the C-MoS_2_/rGO-6 composite exhibits high specific capacity, which hints at the excellent catalytic performance of the defect-rich MoS_2_ towards polysulfide ions.

## Conclusion

Defect-rich porous C-MoS_2_/rGO composites have been successfully fabricated via double modification of MoS_2_ with rGO and amorphous carbon layers. The subsequent annealing at 600 °C enhances the synergistic effect between MoS_2_ and rGO and amorphous carbon and further improves the capability of polysulfide conversion. This also provides an idea for designing other transition-metal dichalcogenide composites for energy storage applications.

## Supporting Information

File 1 Additional experimental data.
