# Supplementary material for: Design and facile synthesis of defect-rich C-MoS2/rGO nanosheets for enhanced lithium–sulfur battery performance
Source: Beilstein J Nanotechnol. 2019 Nov 14;10:2251–60. doi: 10.3762/bjnano.10.217 (PMC6880788; doi:10.3762/bjnano.10.217)
Supplement: File 1 — Additional experimental data. [file Beilstein_J_Nanotechnol-10-2251-s001.pdf]

BEILSTEIN JOURNAL OF NANOTECHNOLOGY

Supporting Information

for

Design and facile synthesis of defect-rich C-MoS_2/rGO nanosheets for enhanced lithium–sulfur battery performance

Chengxiang Tian, Juwei Wu, Zheng Ma, Bo Li, Pengcheng Li, Xiaotao Zu and Xia Xiang

Beilstein J. Nanotechnol. **2019,** *10,* 2251–2260. doi:10.3762/bjnano.10.217

Additional experimental data

License and Terms: This is a supporting information file under the terms of the Creative Commons Attribution License (http://creativecommons.org/licenses/by/4.0). Please note that the reuse, redistribution and reproduction in particular requires that the authors and source are credited.
The license is subject to the *Beilstein Journal of Nanotechnology* terms and conditions: (https://www.beilstein-journals.org/bjnano)

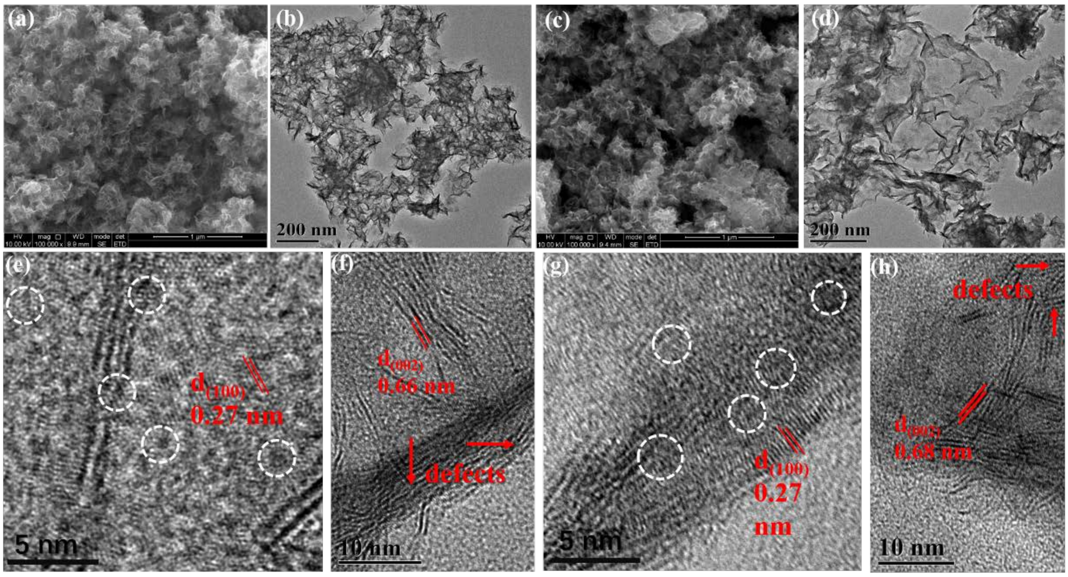

Figure S1: Morphological images of the C-MoS_2/rGO-4 (a) SEM image, (b) TEM image, and (e-f) HRTEM images; Morphological images of the C-MoS_2/rGO-8 (c) SEM image, (d) TEM image, (g-h) HRTEM images.

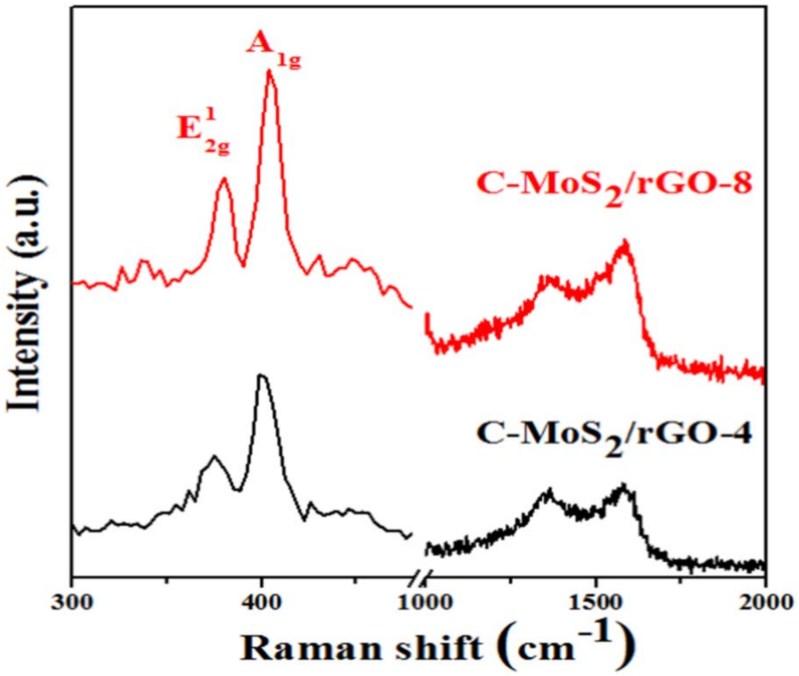

Figure S2: Raman spectra of C-MoS_2/rGO-4 and C-MoS_2/rGO-8 composites.

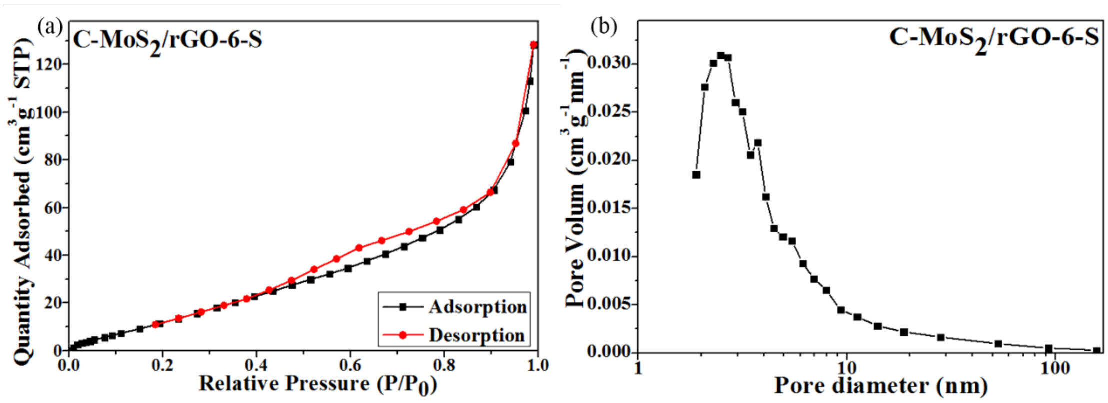

Figure S3: (a) N_2 adsorption–desorption isotherm and (b) corresponding pore size distribution of the C-MoS_2/rGO-6-S composite.

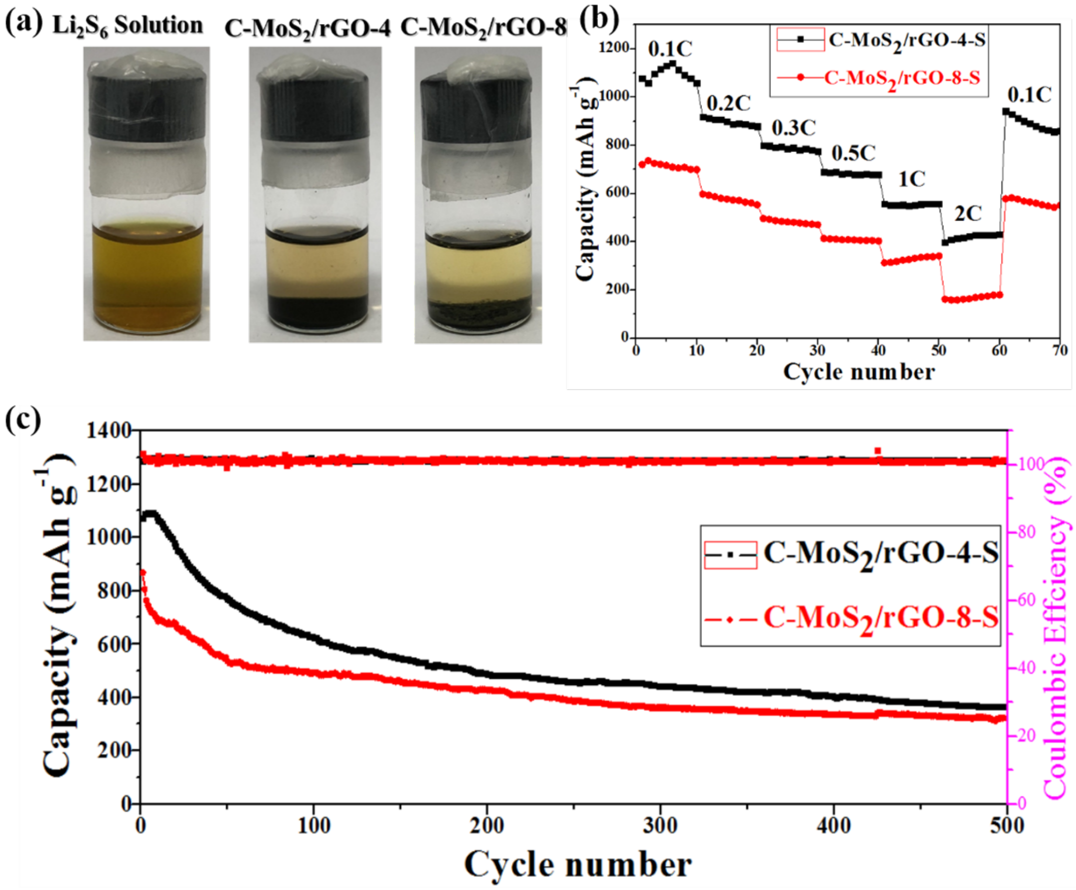

Figure S4: (a) Photos of the Li_2S_6 solution after 12 h of adsorption experiments with the same amount of C-MoS_2/rGO-4 and C-MoS_2/rGO-8 composites. (b, c) Rate performances at different current densities and long-term cycling performance at 0.2C of the C-MoS_2/rGO-4 and C-MoS_2/rGO-8 composites.
